# Reversible Emitting Anti‐Counterfeiting Ink Prepared by Anthraquinone‐Modified *β*‐Cyclodextrin Supramolecular Polymer

**DOI:** 10.1002/advs.202000803

**Published:** 2020-06-15

**Authors:** Lei Chen, Yong Chen, Hong‐Guang Fu, Yu Liu

**Affiliations:** ^1^ College of Chemistry State Key Laboratory of Elemento‐Organic Chemistry Nankai University Tianjin 300071 P. R. China

**Keywords:** anthraquinone photoreduction, cyclodextrin, fluorescent anti‐counterfeiting, fluorescent encryption, supramolecular assembly

## Abstract

Intelligent fluorescent materials have been paid more and more attention due to their wide application in information encryption and anti‐counterfeiting materials. Herein, a supramolecular polymer is constructed through the host–guest interaction of anthraquinone‐modified *β*‐cyclodextrin (AQ‐*β*‐CD) in aqueous solution. Thanks to the hydrophobic microenvironment of the cyclodextrin cavity and the shielding effect on oxygen molecules, the anthraquinone group, as the guest molecule, can rapidly produce 9,10‐anthracenediol (QH_2_) with strong fluorescence by photoreduction. Interestingly, the generated anthracenediol group is still sensitive to oxygen and can be converted to anthraquinone by oxygen. Significantly, aqueous solution of AQ‐*β*‐CD supermolecular polymer is used as emitting ink, which decrypts the information by ultraviolet light and encrypts the information in the air.

## Graphical abstract

Supramolecular polymers with photoreduction properties are constructed from anthraquinone‐modified *β*‐cyclodextrin (AQ‐*β*‐CD) self‐assembled in aqueous solution. Thanks to the hydrophobic microenvironment of the cyclodextrin cavity and the shielding effect on oxygen molecules, the anthraquinone group can produce 9,10‐anthracenediol with strong fluorescence by photoreduction. Moreover, the 9,10‐anthracenediol group is still sensitive to oxygen and can be converted to anthraquinone by oxygen.

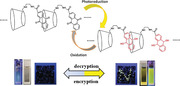

With the rapid development of science and technology, information confidentiality and anti‐counterfeiting become more and more important.^[^ ^1^ ^]^ Among them, intelligent response fluorescent materials are widely developed as anti‐counterfeiting materials due to their unique response properties.^[^ ^2^ ^]^ Under the external stimulation, the fluorescent output of this kind of fluorescent material can be completely changed, which can prevent encrypted information from being read or the data from being forged. In recent years, many intelligent fluorescent materials have been reported and exhibit excellent photophysical behavior under external stimulation.^[^ ^3^ ^]^ Among the numerous external stimuli, photoregulated luminescence has been widely recognized in scientific research due to its advantages of cleanliness, controllability, and non‐pollution of materials.^[^ ^4^ ^]^ The most commonly used method is to transform the different light output states of fluorescent materials by different wavelengths of light radiation.^[^ ^5^ ^]^ However, as information anti‐counterfeiting and encryption materials, being able to encrypt information spontaneously in the air atmosphere is more conducive to prevent information leakage.

Anthraquinone group, as a typical photoactivated group, its photoactivated mechanism has been reported.^[^ ^6^ ^]^ Under ultraviolet light, anthraquinone can undergo photoreduction reaction to produce anthracenediol with strong fluorescence. In the air environment, anthracenediol is oxidized spontaneously to anthraquinone without fluorescence. This type of intelligent response groups has been regarded as ideal candidates for fluorescent anti‐counterfeiting material. However, the process of photoreduction reaction can only take place in deoxidized solution,^[^ ^6b^ ^]^ which greatly limits its development as intelligent fluorescent materials. It is generally reasoned that anthraquinone should be encapsulated in a suitable substrate to build a stable intelligent fluorescent material.^[^ ^7^ ^]^ The suitable substrate matrix needs to meet two conditions: 1) providing a sufficient environment for the efficient phototautomerization of anthraquinone in air condition; and 2) photoreduction products maintain the original oxygen‐sensitive properties. Unfortunately, to the best of our knowledge, anthraquinone as an intelligent fluorescent material has not been reported so far.

Supramolecular polymers with dynamic structures are formed by monomers through directional noncovalent interactions such as electrostatic interaction, metal–ligand interaction, hydrogen bond interaction, *π*–*π* interaction, and host–guest interaction.^[^ ^8^ ^]^ It is considered as one of the ideal ways to build intelligent materials. Cyclodextrin is a kind of ring oligosaccharide with molecular compatibility cavity. Hydrophobic organic molecules can enter the cavity of cyclodextrin to form water‐soluble supramolecular assemblies.^[^ ^9^ ^]^ It is the most widely used macrocyclic molecule in supramolecular chemistry and have been considered as one of the most important building blocks of supramolecular polymer. The supramolecular polymer constructed by cyclodextrins shows biocompatibility, good water solubility, and low toxicity, which are considered to have great application prospects in drug solubilization and delivery.^[^ ^10^ ^]^ For example, Huang and co‐workers used *β*‐cyclodextrin as the host and camptothecin (CPT) as the guest molecule, which was linked by disulfide bond. After the formation of supramolecular polymer, the solubility of CPT was increased by 232 times and inhibits lactone ring opening in physiological environment.^[^ ^9b^ ^]^ In addition, the cavity of cyclodextrin can provide a protective environment for the excited triplet substances to prevent them from being quenched by oxygen molecules or other substances.^[^ ^11^ ^]^ Tian and co‐workers reported that *β*‐cyclodextrin and phosphorescence molecule bromonaphthalene formed supramolecular compound in water and room temperature phosphorescence of bromonaphthalene in water was realized under the protection of cyclodextrin cavity.^[^ ^12^ ^]^ Inspired by the above work, we speculated that *β*‐cyclodextrin may be an ideal matrix for the encapsulation of anthraquinone groups.

Although the inclusion complex of anthraquinone and cyclodextrin has certain water solubility, as a material, the solubility cannot meet the requirements. Therefore, we modify the anthraquinone group on the cyclodextrin by covalent modification to synthesize water‐soluble anthraquinone‐modified *β*‐cyclodextrin (AQ‐*β*‐CD). In aqueous solution, circular dichroism spectrum, Two‐dimensional nuclear magnetic resonance (2D NMR), NMR titration, and transmission electron microscopy (TEM) are used to determine the conformation of AQ‐*β*‐CD. AQ‐*β*‐CD has been proved to be self‐assembled in the form of head‐to‐tail in aqueous solution to form supramolecular polymer. Interestingly, as shown in **Scheme** **1**, AQ‐*β*‐CD aqueous solution can produce obvious fluorescence emission by UV irradiation and this process is confirmed as the photoreduction process of anthraquinone. In addition, photoreduction products can respond to oxygen sensitively. Finally, AQ‐*β*‐CD aqueous solution can be written on filter paper as emitting ink with intelligent response.

**Scheme 1:**
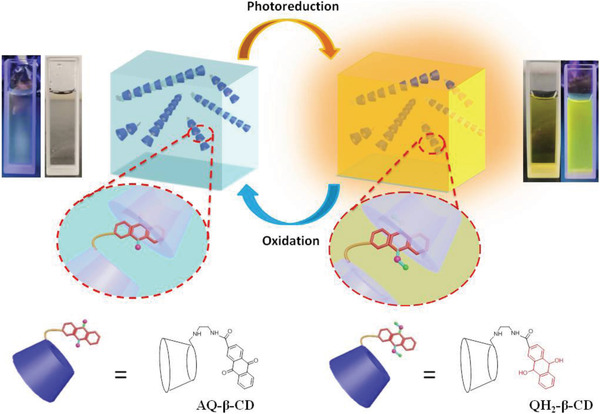
Photoreduction luminescence and oxidation process of AQ‐*β*‐CD supramolecular polymer in aqueous solution.

**Scheme 2:**
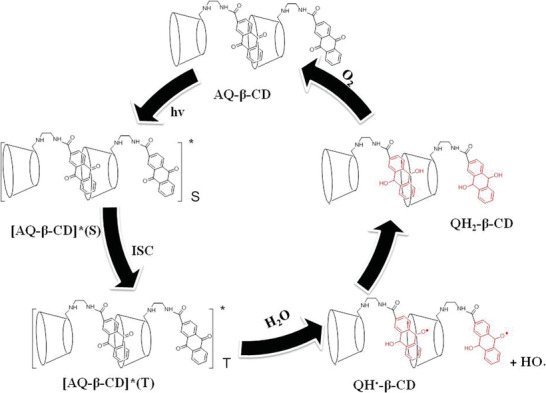
Schematic diagram of photoreduction and oxidation and mechanism of AQ‐*β*‐CD in aqueous solution.

AQ‐*β*‐CD was synthesized by condensation of ethylenediamine‐modified *β*‐cyclodextrin with anthraquinone‐2‐carboxylic acid (Scheme S1, Supporting Information). The assembly mode of anthraquinone group and *β*‐cyclodextrin cavity has been studied in detail. The conformation of anthraquinone group and *β*‐cyclodextrin in aqueous solution was studied by circular dichroism spectroscopy. As shown in Figure S4, Supporting Information, the circular dichroism spectrum of AQ‐*β*‐CD shows a positive cotton peak at 335 nm (^1^La) and a negative cotton peak appears at about 400 nm (^1^Lb). It is speculated that ^1^La belongs to the spectral transition term of carbonyl group and ^1^Lb belongs to the spectral transition term of benzene ring region. According to Kajtr's sector rule on circular dichroism of cyclodextrin inclusion complex,^[^ ^13^ ^]^ we speculate that the anthraquinone group enters the cyclodextrin cavity in an inclined manner. Furthermore, to prove the specific inclusion mode of anthraquinone group in the cyclodextrin cavity, we carried out 2D NMR experiments on AQ‐*β*‐CD. In D_2_O solution, the rotating frame overhauser effect spectroscopy (ROESY) spectrum of AQ‐*β*‐CD shows the obvious nuclear overhauser effect (NOE) correlation peak, providing strong evidence for the inclusion of anthraquinone group in the cavity of cyclodextrin (Figure S5, Supporting Information). In **Figure** **1**a, we can easily find the NOE cross‐peaks between the H_a_ and H_b_ protons of anthraquinone group and H_3,5_ protons of cyclodextrin (peaks B and peaks D). In addition, the NOE cross‐peaks between the H_e_ and H_d_ protons of anthraquinone group and H_3,5_ protons of cyclodextrin can also be observed (peaks A and peaks C), although it is very weak, which means that the H_e_ and H_d_ protons are far away from the cyclodextrin cavity. However, no NOE cross‐peaks between H_c_ protons of anthraquinone group and H_3,5_ protons of cyclodextrin could be observed. It can be inferred from the above results that the anthraquinone group enters the cavity of *β*‐cyclodextrin in an inclined manner as shown in the Figure 1a inset.

**Figure 1:**
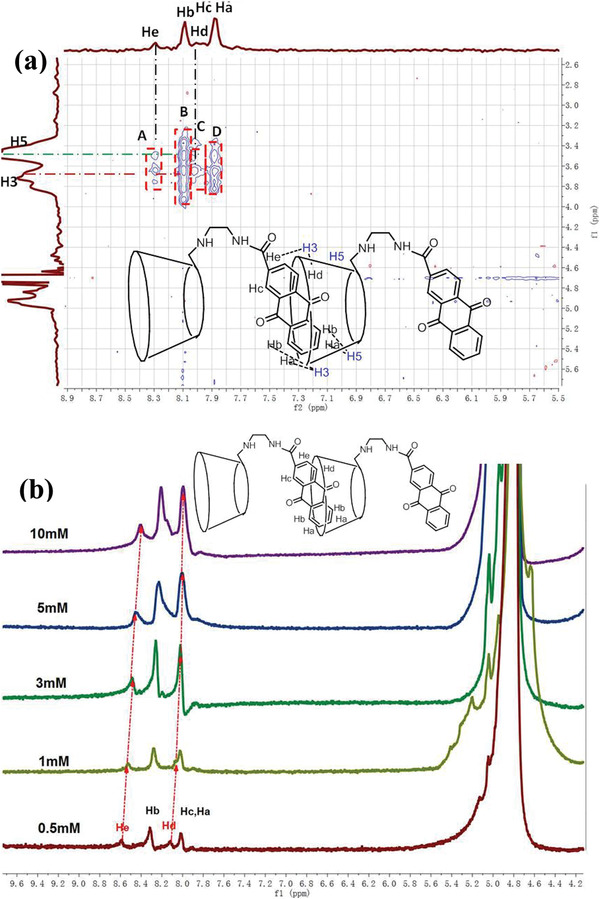
a) Part of 2D NOESY (400 MHz, D_2_O, 25 °C) spectral of AQ‐*β*‐CD. Inset: schematic diagram of anthraquinone group assembly in cyclodextrin cavity ((AQ‐*β*‐CD) = 1.0 × 10^−3^ m). b) ^1^H NMR spectra of AQ‐*β*‐CD at different concentrations. Inset: schematic diagram of anthraquinone group assembly in cyclodextrin cavity ((AQ‐*β*‐CD) = 0.5 × 10^−3^, 1.0 × 10^−3^, 3 × 10^−3^, 5 × 10^−3^, and 10 × 10^−3^ m).

Cyclodextrins with hydrophobic groups have different self‐assembly forms in aqueous solution through the synergistic action of various non‐covalent bond forces.^[^ ^14^ ^]^ The self‐assembly constant (*K*_a_) of cyclodextrin derivatives in aqueous solution can be determined by NMR titration and the self‐assembly behavior of cyclodextrin derivatives in aqueous solution can be inferred. Therefore, we tested the ^1^H NMR spectra of AQ‐*β*‐CD with different concentrations (0.5 × 10^−3^, 1.0 × 10^−3^, 3 × 10^−3^, 5 × 10^−3^, and 10 × 10^−3^ m) (Figure 1b). The relationship between chemical shift and concentration of H_e_ on anthraquinone group was measured. The constant δ_mon_ and δ_agg_ were obtained by fitting the above parameters with the software (Figures S6 and S7, Supporting Information). Assuming dimer, *K*_a_ value was calculated by Equation (S2), Supporting Information. The *K*_a_ values of AQ‐*β*‐CD are listed in Table S1, Supporting Information, along with the complex stability constants (*K*_s_) of native *β*‐cyclodextrins with the anthraquinone‐2‐carboxylic acid. By comparing *K*_a_ and *K*_s_, it can be found that there is no significant difference between them. This result indicated that the anthraquinone group on the modified *β*‐cyclodextrins penetrates into another *β*‐cyclodextrin cavity to form a head‐to‐tail dimer complex, while the anthraquinone group on the *β*‐cyclodextrins that are penetrated into the enterprising substituents is certainly not involved in the formation of this complex.^[^ ^15^ ^]^ After dimer formation, it further extends into linear supramolecular polymer. Unfortunately, the *K*_s_ is small enough to test for specific molecular weights. However, 2D diffusion‐ordered NMR spectroscopy investigations indicated that the measured weighted average diffusion coefficients (*D*) considerably decreased to 1.99 × 10^−10^ m^2^ s^−1^ (Figure S8, Supporting Information). It is confirmed that AQ‐*β*‐CD forms supramolecular polymer in aqueous solution. Similarly, the linear structure was also observed by TEM, which is the direct evidence for the formation of supramolecular polymers (**Figure** **2**).

**Figure 2:**
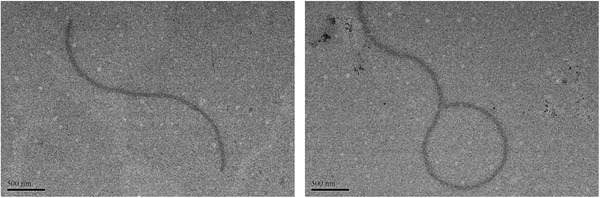
TEM images of AQ‐*β*‐CD ((AQ‐*β*‐CD) = 1 × 10^−3^ m).

With the formation of supramolecular polymer in aqueous solution, the properties of photoactivation luminescence have been explored in detail. As shown in the **Figure** **3**a, the aqueous solution of AQ‐*β*‐CD supramolecular polymer with a concentration of 1 × 10^−3^ m was exposed to the UV light. After 18 min of UV radiation, the aqueous solution of AQ‐*β*‐CD changes from colorless transparent solution to light yellow solution and the fluorescence emission began to appear at 550 nm and the intensity gradually increased. When the fluorescence intensity is stable, the fluorescence lifetime is 5.10 ns and the quantum yield is 3.40% (Figure S9, Supporting Information). In addition, it can be found through UV–visible absorption spectrum that the absorption of 330 nm gradually decreases while a new absorption appears at about 380 nm and increases with the extension of UV irradiation (Figure 3b). That means that something new is being created in the process. Interestingly, this fluorescence is very sensitive to oxygen. After bubbling air in aqueous solution, the aqueous solution returns to a colorless and transparent state and the fluorescence disappears immediately (Figure 3c). This kind of fluorescence reciprocating process of aqueous solution can be repeated at least six times without fluorescence damage (Figure 3d). Moreover, a comparison of the mass spectrometry data shows that the molecular weight of AQ‐*β*‐CD has no obvious change before and after six cycles of UV light illumination (Figure S3, Supporting Information). In addition, no room temperature phosphorescence emission of this system in aqueous solution can be observed either before or after illumination.

**Figure 3:**
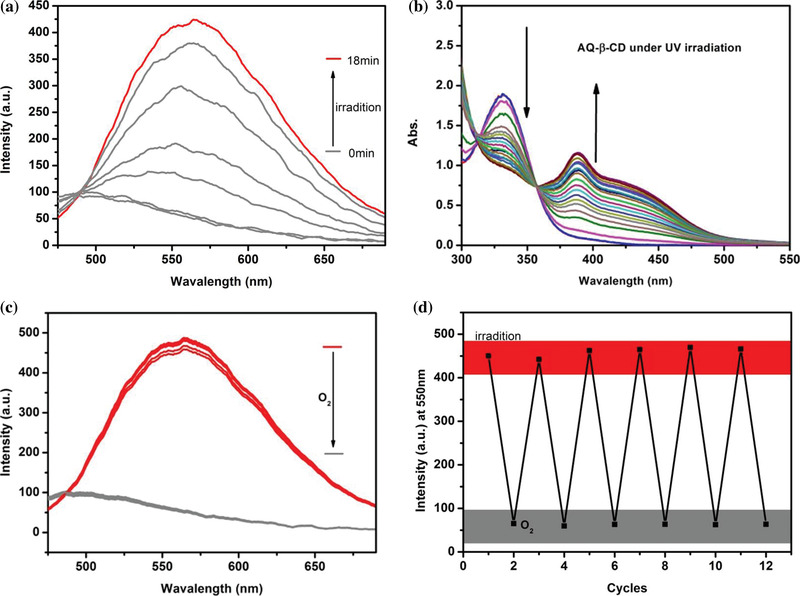
a) Emission spectra of AQ‐*β*‐CD aqueous solution upon irradiation with UV light ((AQ‐*β*‐CD) = 1.0 × 10^−3^ m). b) UV–vis absorption spectrum of AQ‐*β*‐CD aqueous solution upon irradiation with UV light ((AQ‐*β*‐CD) = 1.0 × 10^−3^ m). c) Emission spectra of AQ‐*β*‐CD aqueous solution upon alternating UV (365 nm, 18 min) and bubbling air at 298 K. d) Fluorescence transformation repeatability test.

In order to explore the mechanism of photoactivation of the aqueous solution of AQ‐*β*‐CD supramolecular polymer, the infrared absorption spectra of the AQ‐*β*‐CD before and after irradiation were measured in situ. In the **Figure** **4**a, the absorption peak of anthraquinone carbonyl is between 1687 and 1665 cm. The carbonyl signal of anthraquinone almost disappeared after irradiation by UV for enough time, which means that carbonyl is not included in the product after irradiation. According to the infrared results, it is inferred that the photoreduction of anthraquinone occurs under the condition of illumination.

**Figure 4:**
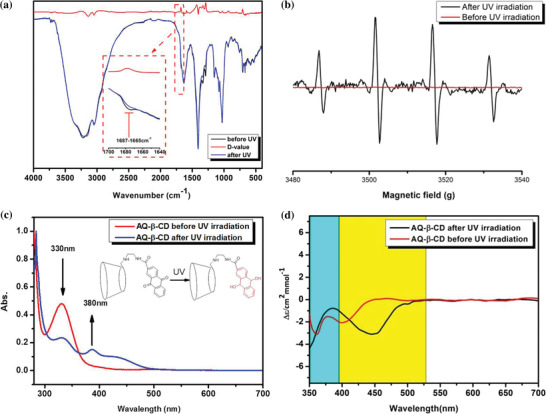
a) FTIR spectra of AQ‐*β*‐CD before and after UV irradiation. b) EPR spectra of AQ‐*β*‐CD before and after UV irradiation. c) UV–vis spectra of AQ‐*β*‐CD before and after UV irradiation. d) The circular dichroism spectra of AQ‐*β*‐CD before and after UV irradiation.

The photoreduction of anthraquinone group in AQ‐*β*‐CD was further confirmed by electron paramagnetic resonance (EPR) and UV–vis absorption spectrometer (Figure 4b,c). A total of 20 µL 1 M free radical‐trapping agent, DMPO, was added to AQ‐*β*‐CD aqueous solution before irraditaion. The EPR signal was tested after UV irradiation. The characteristic EPR spectra for the DMPO‐OH adducts with a 1:2:2:1 quadruple signal with aN = aH = 14.9 G were observed in Figure 3b. This paramagnetic signal means that irradiation produces hydroxyl radicals. In addition, the paramagnetic signal was not detected in EPR without the addition of trapping agent, which means that the final yellow product is not free radical (Figure S10, Supporting Information). Furthermore, UV–vis absorption spectrometer was used to monitor the absorption change during irradiation process. The absorption peak of anthraquinone chromogenic group decreased at about 330 nm, while a new group of UV absorption peaks appeared at about 380 and 450 nm. According to the special absorption of UV spectrum, it is speculated that the strong fluorescence material is QH_2_ produced by photoreduction of anthraquinone.^[^ ^6b^ ^]^ The production of QH_2_ and hydroxyl radicals can further explain that the photoactivation process of AQ‐*β*‐CD supramolecular polymer comes from the photoreduction of anthraquinone group. In addition, the circular dichroism spectrum of AQ‐*β*‐CD shows that the chromophore has obvious circular dichroism signal before and after irradiation, which indicates that the photoreduction process of chromophore is always in the cavity of *β*‐cyclodextrin (Figure 4d).

To further study the role of cyclodextrin cavity in the photoreduction of anthraquinone group, a series of control experiments were carried out by UV–vis spectrum and fluorescence spectra. On the one hand, anthraquinone‐2‐carboxylic acid was dissolved in alkaline aqueous solution, and its UV absorption and fluorescence emission before and after irradiation in air condition were measured. The characteristic absorption peak of QH_2_ can neither be detected by UV–vis spectrum nor seen by fluorescence emission (Figure S11a,b, Supporting Information). On the other hand, adamantane amine was added to AQ‐*β*‐CD aqueous solution to make adamantane interact with cyclodextrin cavity to occupy the cavity position. After irradiation by UV in air condition, the characteristic peaks of QH_2_ were also unavailable (Figure S11c,d, Supporting Information). The results show that anthraquinone group enters the cavity of cyclodextrin, which plays a unique role in photoreduction.

Based on the above results, we speculated the mechanism of photoactivation luminescence of the AQ‐*β*‐CD in aqueous solution as follows. As shown in Scheme 2, First, the anthraquinone group interacts with the *β*‐cyclodextrin's secondary surface in an inclined way, that is to say, at least one side of the carbonyl group on anthraquinone is in the hydrophobic cavity of *β*‐cyclodextrin. Second, along with the irradiation, after the anthraquinone group absorbs photons, it transits from ground state to excited singlet state ((AQ‐*β*‐CD)*_S_), and then transits to excited triplet state ((AQ‐*β*‐CD)*_T_) through system crossing. This process can be done quickly under UV light, because the hydrophobic cavity of cyclodextrin can accelerate the photoreduction process of anthraquinone carbonyl in the cavity.^[^ ^16^ ^]^ The excited triplet‐state anthraquinone group can extract hydrogen atom from aqueous solution to form semiquinone radical (QH^•^‐*β*‐CD) and hydroxyl radical pair (OH^•^). Third, after the carbonyl group in the cavity of *β*‐cyclodextrin transforms to the semiquinone radical, the hydrophobic cavity can prevent semiquinone radical from being scavenged by oxygen. Finally, the QH^•^‐*β*‐CD undergoes further disproportionation to form QH_2_‐*β*‐CD in the cavity of *β*‐cyclodextrin with yellow fluorescence. Herein, the hydrogen bonds between the anthracenediol arm and the hydroxyl groups of *β*‐CD cavity not only enhance the rigidity of the assembly but also protect the emitting anthracenediol center from the attack of bulk water, which jointly enhances the emitting process. However, only one part of anthracenediol molecule is embedded in the cavity of *β*‐cyclodextrin, Therefore, QH_2_‐*β*‐CD can be transformed into AQ‐*β*‐CD by oxygen.

Emitting ink with intelligent response has important applications in anti‐counterfeiting materials and information security. Considering the unique photoreduction luminescence and oxygen response properties, AQ‐*β*‐CD supramolecular polymers have great potential for information encryption and decryption. AQ‐*β*‐CD aqueous solution was prepared with a concentration of 1 × 10^−3^ m. The solution was then used in writing it as ink on the filter paper printed with 2D code and drying it naturally to get 2D code with anti‐counterfeiting pattern. It is worth noting that AQ‐*β*‐CD aqueous solution is colorless and transparent. After writing and drying, the pattern cannot be observed under natural light and UV light (**Figure** **5**), which is crucial for information anti‐counterfeiting and encryption. The interpretation of encrypted information is carried out by photoreduction AQ‐*β*‐CD supramolecular polymers under the illumination of UV lamp. About 5 min later, the pattern N emits yellow fluorescence under UV lamp and the pattern turns yellow and can be observed with the naked eye (Supporting Information II). Interestingly, although the pattern is very sensitive to oxygen, it can still be kept in the air for more than 90 min, which is conducive to data reading and writing. After 150 min, the pattern disappears completely, which is conducive to data confidentiality. The rate of pattern disappearance can be changed by controlling the amount of oxygen in the air (**Figure** **6**).

**Figure 5:**
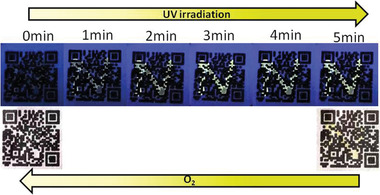
Light response process and oxygen‐sensitive process of AQ‐*β*‐CD supramolecular polymers as intelligent emitting ink.

**Figure 6:**
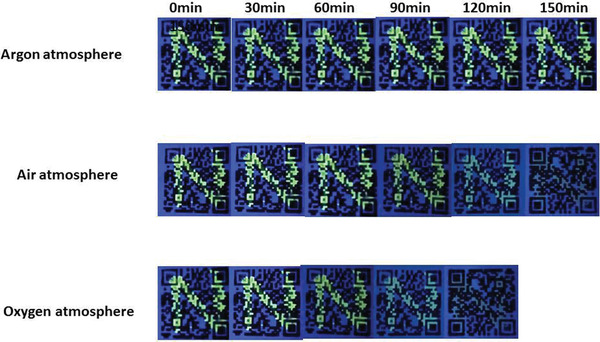
Oxygen‐sensitive fluorescence and its dependence on oxygen.

In summary, we have successfully constructed a supramolecular polymer system in aqueous solution by anthraquinone‐modified *β*‐cyclodextrin. Among them, *β*‐cyclodextrin as the host molecule and anthraquinone as the guest molecule enter the hydrophobic cavity of *β*‐cyclodextrin to form the head‐to‐tail supramolecular linear polymer. Thanks to the protection of cyclodextrin cavity to anthraquinone group, anthraquinone group can be transformed into hydroquinone group with strong fluorescence by photoreduction under UV light. After a contact with oxygen, the hydroquinone in the cavity of *β*‐cyclodextrin can be transformed into the original anthraquinone group. As a result, this unique fluorescence response process enables the AQ‐*β*‐CD supramolecular polymer to be used as an intelligent emitting ink for anti‐counterfeiting and encryption processes. We believe that our strategy will provide a new way to construct intelligent response materials in the future.

## Conflict of Interest

The authors declare no conflict of interest.

## Supporting information

Supporting Information

Supplemental Movie 1
