# Supplementary material for: Reversible Emitting Anti‐Counterfeiting Ink Prepared by Anthraquinone‐Modified β‐Cyclodextrin Supramolecular Polymer
Source: Adv Sci (Weinh). 2020 Jun 15;7(14):2000803. doi: 10.1002/advs.202000803 (PMC7375224; doi:10.1002/advs.202000803)
Supplement: Supplementary file 1 — Supporting Information [file ADVS-7-2000803-s001.pdf]

Supporting Information

for *Adv. Mater.*, DOI: 10.1002/adma.((please add manuscript number))

Reversible emitting anti-counterfeiting ink prepared by anthraquinone modified β-cyclodextrin supramolecular polymer

*Lei Chen, Yong Chen, Hong-Guang Fu and Yu Liu**

Table of Contents

I. Experimental section

II. Measurements

Ⅲ. Figures

Scheme S1. Synthesis route of AQ-β-CD

Figure S1. ^{1}H NMR (400 MHz, D_2O, 25℃) spectrum of AQ-β-CD.

Figure S2. ^{13}C NMR (101 MHz, D_2O, 25℃) spectrum of compound AQ-β-CD.

Figure S3. (a) ESI Mass Spectrometry of AQ-β-CD;(b) ESI Mass Spectrometry of AQ-β-CD after six cycles of UV light

Figure S4. the circular dichroism spectra of AQ-β-CD and (inset) Schematic diagram of anthraquinone group assembly in cyclodextrin cavity ([AQ-β-CD]=1.0mM)

Figure S5. 2D NOESY (400 MHz, D_2O, 25 ^oC) spectral of AQ-β-CD

Figure S6. (a) Plot of chemical shift (δobs) vs the concentration of AQ-β-CD for δmon. (b) Plot of chemical shift (δobs) vs the reciprocal of concentration of AQ-β-CD for δagg. Dots are experimental data. Curve is best fit.

Figure S7. Plot from ^{1}H NMR data of AQ-β-CD as a function of concentration to determine the aggregation equilibrium constant. The solid circles are the experimental data points, and the line is the theoretical curve based on the calculated values from eq 2.

Table S1. Self-aggregation constant (K_a) and bonding constant (K_s) of host-guest inclusion compound at 25°C in aqueous solution

Figure S8. 2D DOSY spectrum (400 MHz, 298 K, D2O) of AQ-β-CD ([AQ-β-CD] = 10 mM).

Figure S9. The fluorescence lifetime of AQ-β-CD after UV irradiation ([AQ-β-CD] = 1 mM)

Figure S10. ESR spectra of AQ-β-CD wthout radical trapping agent after UV irradiation.

Figure S11. (a) UV/Vis spectra of anthraquinone-2-carboxylic acid before and after UV irradiation; (b) Emission spectra of anthraquinone-2-carboxylic acid before and after UV irradiation; (c) UV/Vis spectra of AQ-β-CD and adamantane before and after UV irradiation; (d) Emission spectra of AQ-β-CD and adamantane before and after UV irradiation.

REFERENCES

Experimental Procedures

Measurements

All solvents and reagents were commercially available and used without further purification unless noted otherwise. NMR spectra were recorded on an Ascend 400 MHz instrument. Mass spectra were performed on a LCQ-Adantage. Absorption spectra were record on[2] a Thermo Fisher Scientific EVO300 PC spectrophotometer in a conventional rectangular quartz cell (10×10 ×45 mm) at 25 °C. Fluorescence spectra were measured in a conventional rectangular quartz cell (10 × 10 × 45 mm) on a JASCO FP-750 spectrometer equipped with a constant temperature water bath. Circular dichroism spectra were collected on a spectropolarimeter in a light path 10 mm or 1 mm quartz cell. Transmission Electron Microscope (TEM) measurements were recorded on a high-resolution TEM (Tecnai G2 F20 microscope, FEI) equipped with a CCD camera (Orius 832, Gatan) operating at an accelerating voltage of 200 kV. The UV irradiation experiment was carried out ultraviolet portable lamp (6W, radiation distance is 10cm).

Synthesis and characterization of products

Ethylenediamine-modified β-CDs were synthesized according to previous reports[1] . Anthraquinone-2-Carboxylic acid, 1-(3-Dimethylaminopropyl)-3-ethylcarbodiimide hydrochloride (EDC.HCl), N-Hydroxysuccinimide (NHS) were dissolved in dry DMF at 0℃.Then, Ethylenediamine-modified β-CDs were dissolved in dry DMF and dripped into the above solution. The mixture was stirred roughly at 0 °C for 1h, then at room temperature for 18h, and concentrated in vacuo and drop into acetone to precipitate yellowish to obtain precipitation of crude product. Crude product was further purified by column chromatography. Light pink powder was obtained from freeze-dried product with a yield of 30%.1H NMR (D_2O, ppm) : δ2.3-2.4 (m, 4H, H of aminoethylamino), 3.3-4.0 (m, 42H, H of C–2, C–3, C–4, C–5, C–6), 4.9-5.0 (s, 7H, H of C–1), 7.1-8.4 (m, 7H, H of anthraquinone). . ESI -MS: calcd for [M+H], $C_{59}H_{83}N_2O_{37}$, 1411.276.

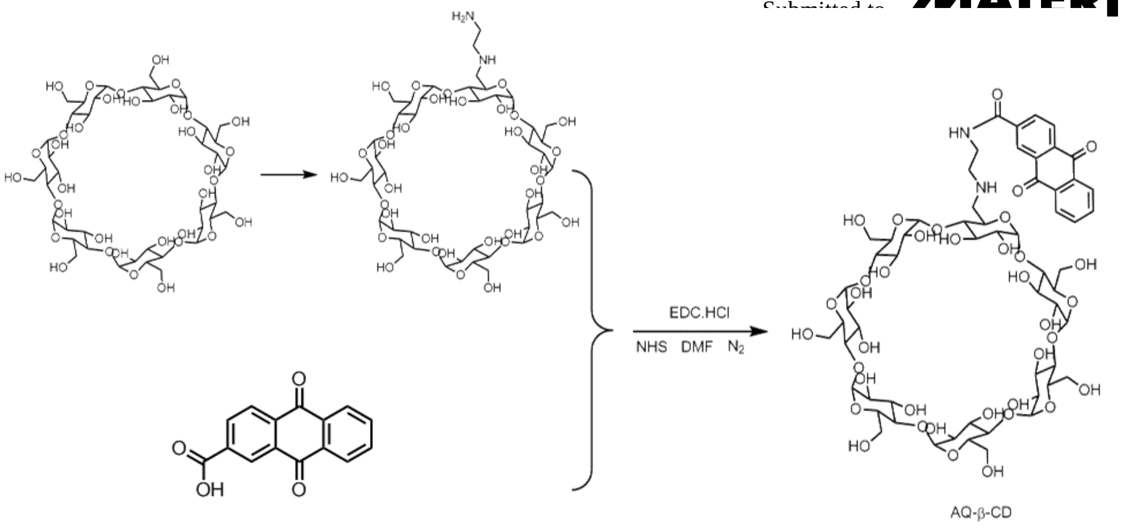

Scheme S1. Synthesis route of AQ-β-CD

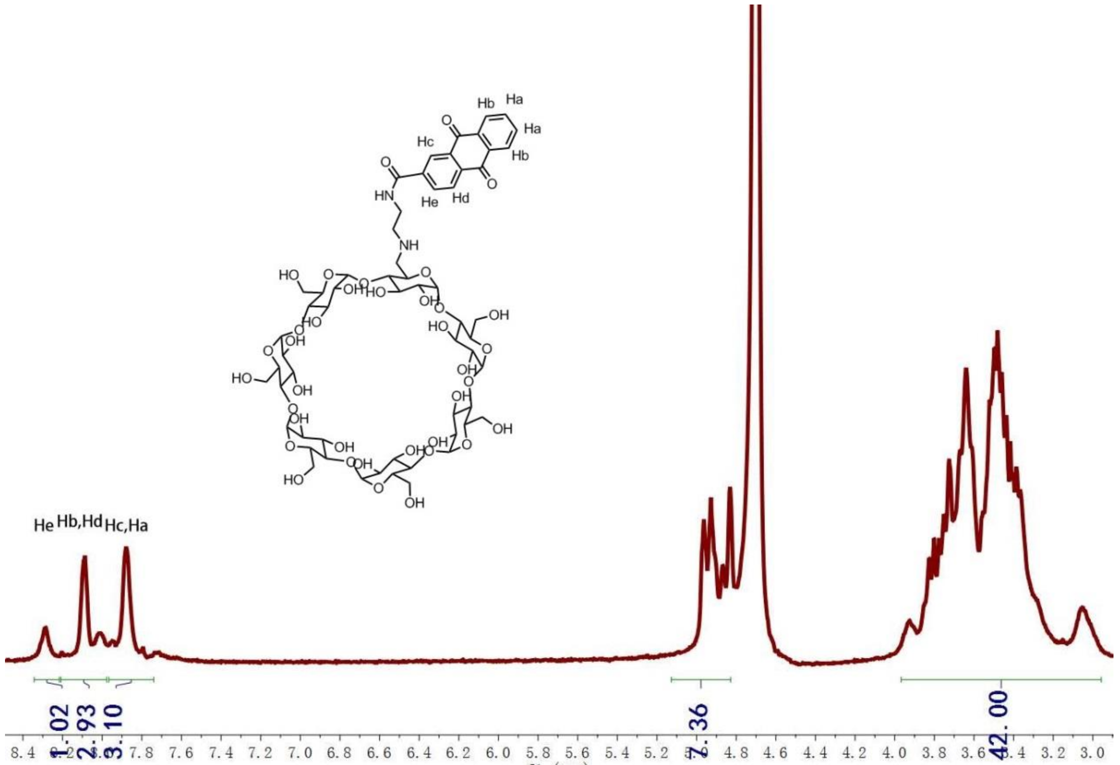

Figure S1. ^{1}H NMR (400 MHz, D_2O, 25℃) spectrum of AQ-β-CD.

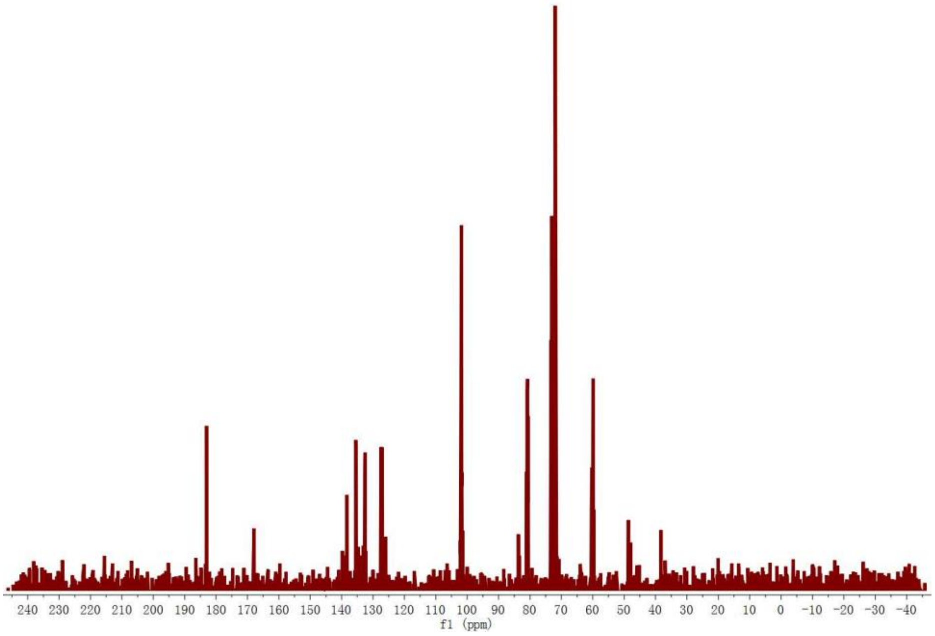

Figure S2. ^{13}C NMR (101 MHz, D_2O, 25℃) spectrum of compound AQ-β-CD.

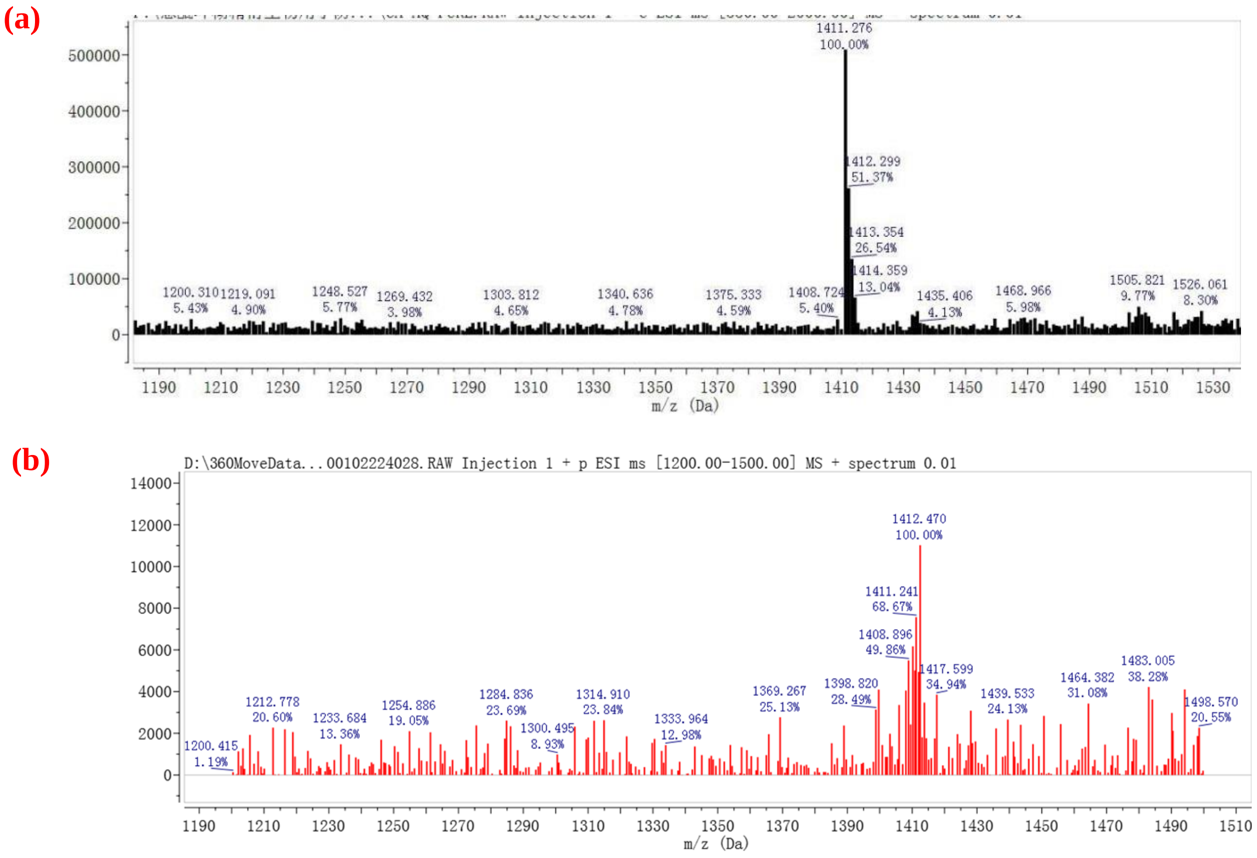

Figure S3. (a) ESI Mass Spectrometry of AQ-β-CD; (b) ESI Mass Spectrometry of AQ-β-CD after six cycles of UV light

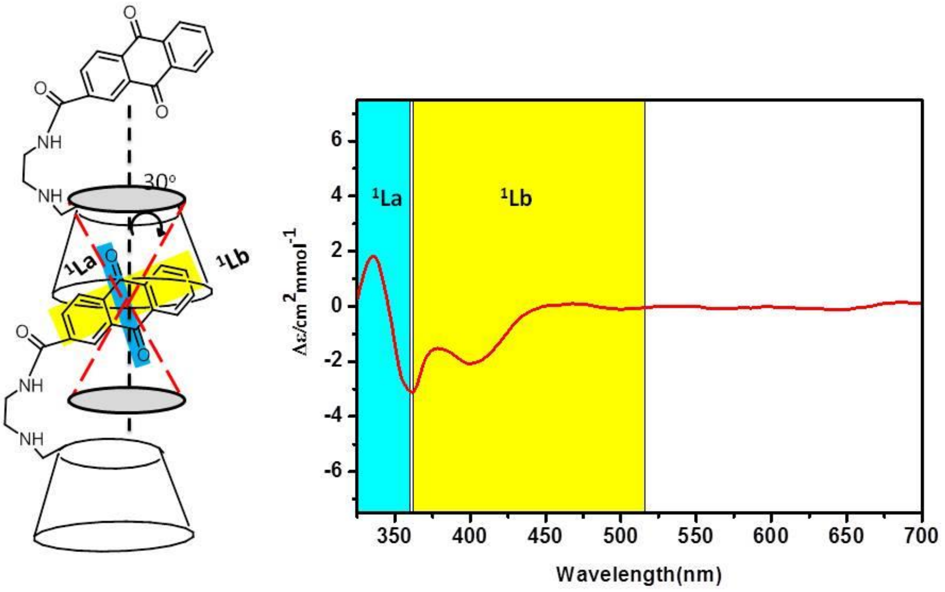

Figure S4. the circular dichroism spectra of AQ-β-CD and (inset) Schematic diagram of anthraquinone group assembly in cyclodextrin cavity ([AQ-β-CD]=1.0mM)

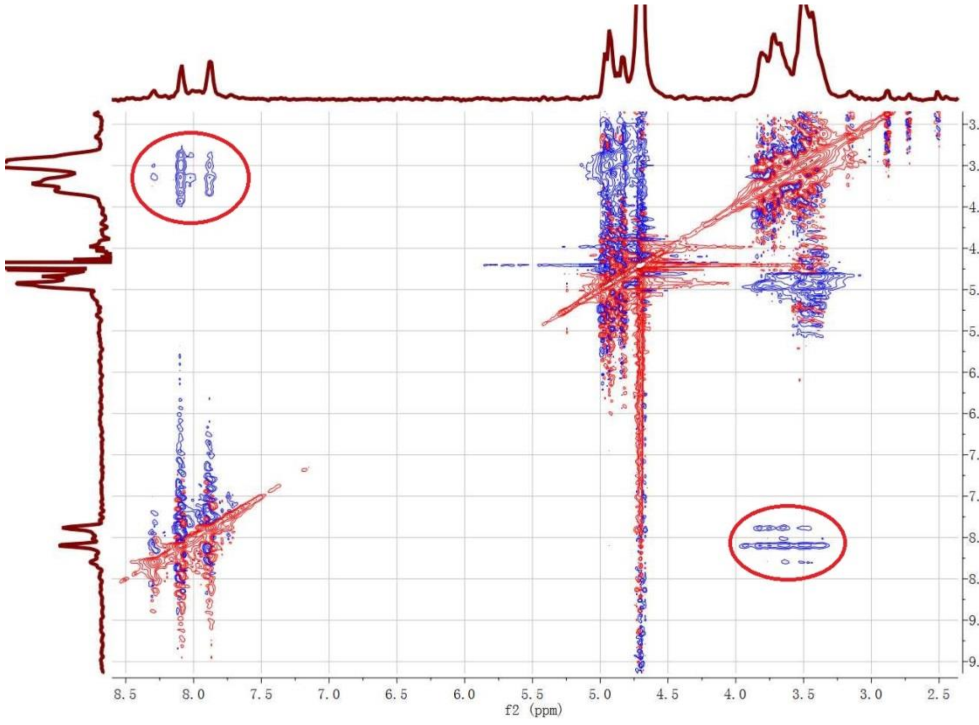

Figure S5. 2D NOESY (400 MHz, D_2O, 25 ^oC) spectral of AQ-β-CD

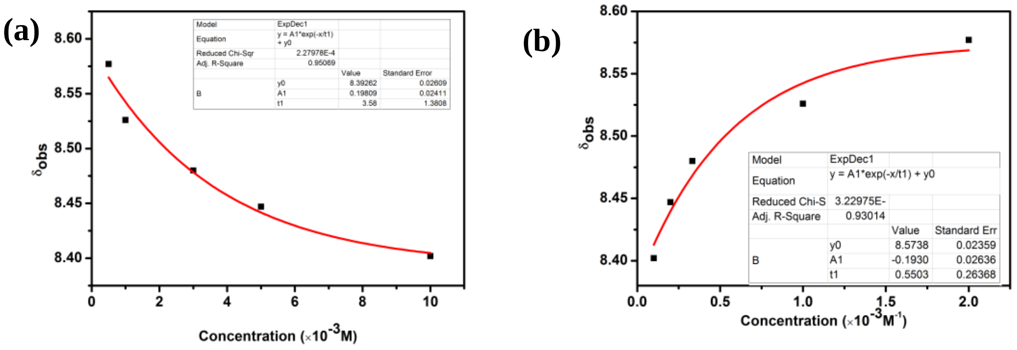

Figure S6. (a) Plot of chemical shift (δobs) vs the concentration of AQ-β-CD for δmon. (b) Plot of chemical shift (δobs) vs the reciprocal of concentration of AQ-β-CD for δagg. Dots are experimental data. Curve is best fit.

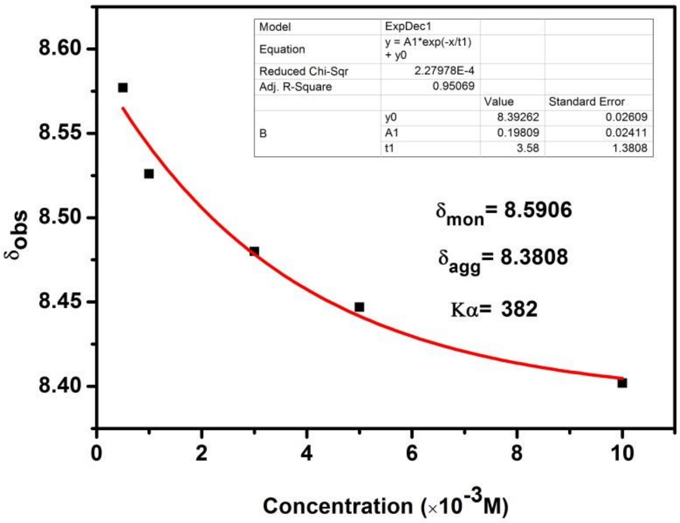

Figure S7. Plot from ^1H NMR data of AQ-β-CD as a function of concentration to determine the aggregation equilibrium constant. The solid circles are the experimental data points, and the line is the theoretical curve based on the calculated values from eq 2.

The NMR titration is a convenient method for determining the self-association constant K_a and aggregation number n of modified β-CD by eq 1:

$$\ln(\delta_{mon} - \delta_{obs})\, C_{tot} = n \ln(\delta_{obs} - \delta_{agg})\, C_{tot} + \ln K_a + \ln n - (n-1)\ln(\delta_{mon} - \delta_{agg}) \quad (1)$$

C_{tot} refers to the total concentration of AQ-β-CD and δ_{mon} and δ_{agg} the extrapolated values of the monomer and aggregate, respectively. By substituting n with 2 in eq 1, we obtain the equation for monomer-dimer equilibrium:

$$\delta_{obs} = \delta_{dimer} + \{(\delta_{mon} - \delta_{dimer})[(-1) + (1 + 8K_a C_{tot})^{1/2}]/(4K_a C_{tot}) \quad (2)$$

Table S1. Self-aggregation constant (K_a) and bonding constant (K_s) of host-guest inclusion compound at 25°C in aqueous solution

Compound/Complexation	K_a/ K_s(M^{-1})	Method	Raf
AQ-β-CD	382	NMR	This work
9,10-anthraquinone-β-CD	135	Cal	[2]

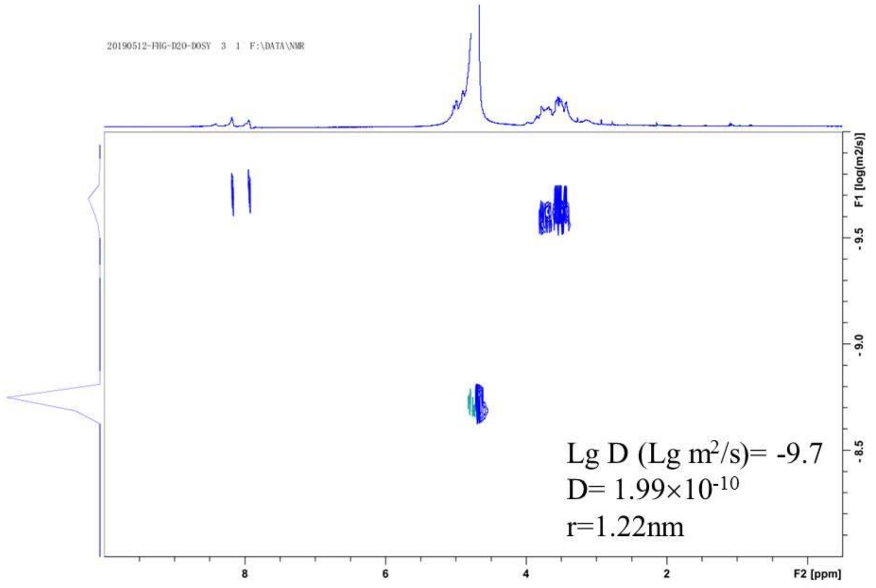

Figure S8. 2D DOSY spectrum (400 MHz, 298 K, D_2O) of AQ-β-CD ([AQ-β-CD] = 10 mM).

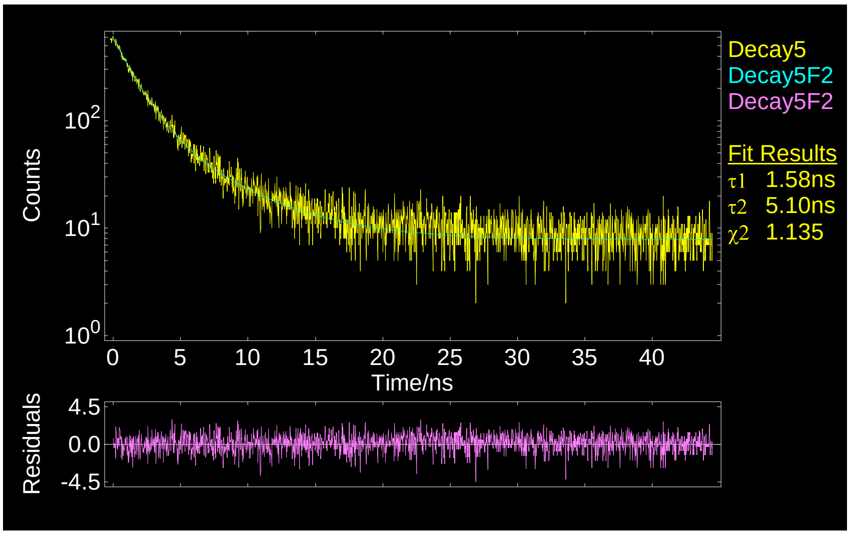

Figure S9. The fluorescence lifetime of AQ-β-CD after UV irradiation ([AQ-β-CD] = 1 mM)

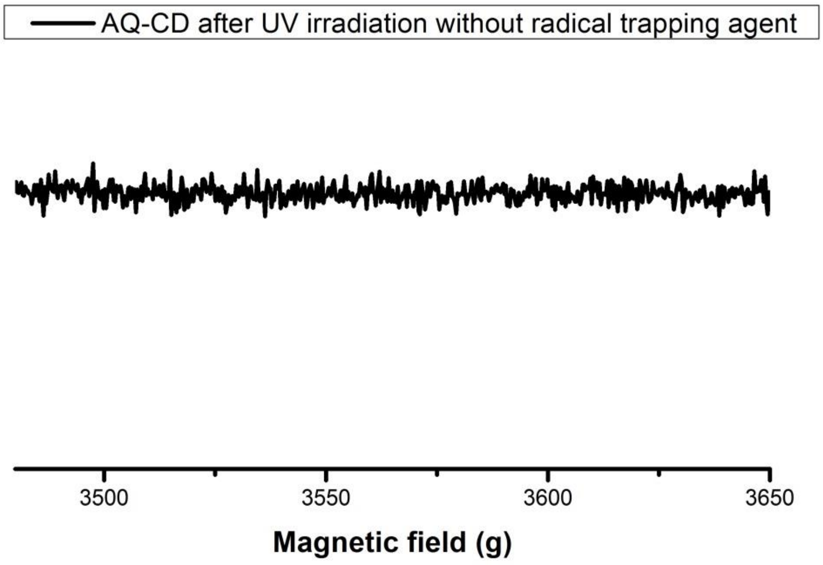

Figure S10. ESR spectra of AQ-β-CD wthout radical trapping agent after UV irradiation.

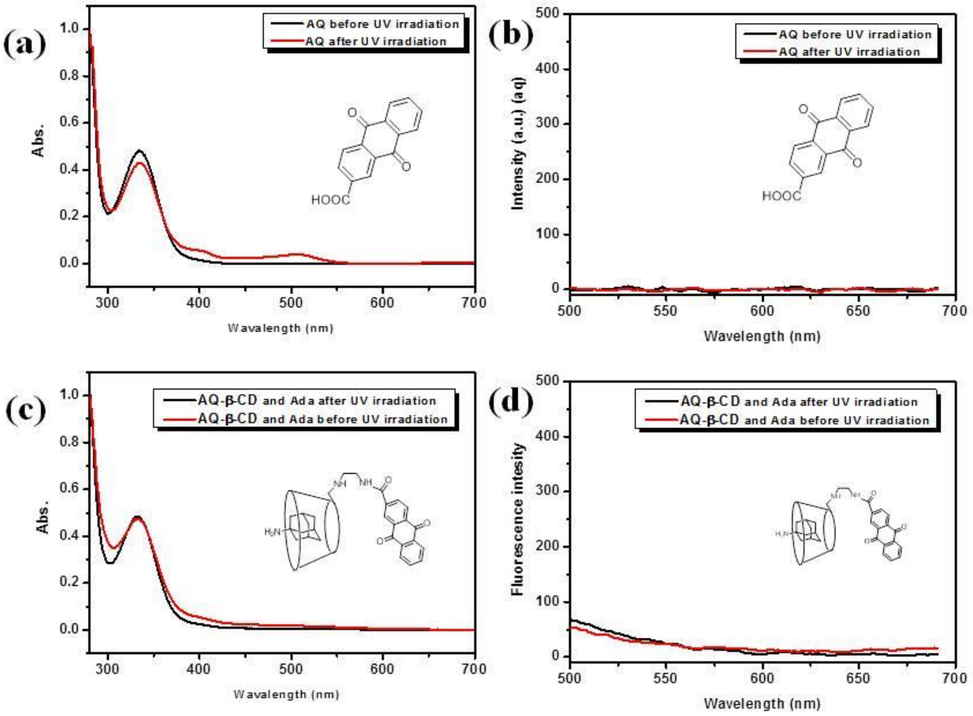

Figure S11. (a) UV/Vis spectra of anthraquinone-2-carboxylic acid before and after UV irradiation; (b) Emission spectra of anthraquinone-2-carboxylic acid before and after UV irradiation; (c) UV/Vis spectra of AQ-β-CD and adamantane before and after UV irradiation; (d) Emission spectra of AQ-β-CD and adamantane before and after UV irradiation.

References

[1] Y. Zhang, L. Liang, Y. Chen, X.M. Chen , Y. Liu, *Soft Mater* **,2019**, *15*, 73.

[2] H. M, Jiang, H.Yang, *J. Hubei Univ*., **2014**, *36*, 90.
